# Spatial correlation effect of haze pollution in the Yangtze River Economic Belt, China

**DOI:** 10.1371/journal.pone.0311574

**Published:** 2024-10-30

**Authors:** Zihai Fang, Zuhan Liu, Yuanhao Hu

**Affiliations:** 1 School of Information Engineering, Nanchang Institute of Technology, Nanchang, China; 2 Jiangxi Province Key Laboratory of Smart Water Conservancy, Nanchang, China; NED University of Engineering and Technology, PAKISTAN

## Abstract

With the rapid development of industry, haze pollution has become an urgent environmental problem. This study innovatively utilizes network-based methods to investigate the spatial correlation effects of haze pollution transmission between urban clusters in the Yangtze River Economic Belt. A spatial correlation network of haze pollution in the Yangtze River Economic Belt was constructed using 328 urban meteorological data collection points as research samples, and its structural characteristics were examined. Main findings are as follows: (1) The spatial correlation network of PM_2.5_ in the Yangtze River Economic Belt urban agglomeration exhibits typical network structural characteristics: obvious spatial correlation within the network. (2) Chengdu, Chongqing, Wuhan, Nanchang, Yichang, Changsha and Yueyang are located at the center of the spatial network. They have more receiving and sending relationships. (3) 36 cities can be divided into four types: bilateral overflow, net beneficiary, net overflow and broker. Each type has different functional characteristics and linkage effects in the network. (4) Haze pollution positively correlates with the city’s synergistic development capacity and urbanization rate, the higher the city’s development level and the higher the Urbanization rate, the stronger its haze pollution capacity. This study provides new insights into the study of the spatial correlation and impact of haze pollution.

## 1. Introduction

In recent years, with the acceleration of industrialization and urbanization, environmental problems in China have become increasingly prominent, especially the air pollution problem characterized by haze. At the beginning of 2013, China experienced a persistent and widespread severe haze problem, affecting over 8 million people. The direct economic losses caused by this hazing incident were about 23 billion yuan, which is considered the most serious air pollution incident in China since the last century [1, 2]. Gaining economic growth at the expense of the environment is no longer desirable, so environmental protection has emerged as the highest priority among current tasks [3].

The Yangtze River Economic Belt spans eastern, central and western China, encompassing a population and economic output that account for "half of the country." It serves as a vital ecological security barrier for China. The belt comprises 11 provinces and cities: Shanghai, Jiangsu, Zhejiang, Anhui, Jiangxi, Hubei, Hunan, Chongqing, Sichuan, Yunnan and Guizhou Province. It’s noteworthy that Yunnan and Guizhou Province are excluded from this study due to their notably good air quality compared to other regions in the belt. On November 27, 2023, the Political Bureau of the Central Committee of the Communist Party of China convened a meeting to review the Opinions on Several Policies and Measures to Further Promote the high-quality Development of the Yangtze River Economic Belt. The meeting emphasized that the high-quality development of the belt hinges on the ecological well-being of the Yangtze River Basin. The economic growth of China has been significantly driven by the Yangtze River Economic Belt, making it a crucial national strategy [4]. To contribute to the long-term construction and high-quality development of the Yangtze River Economic Belt, this study aims to investigate the characteristics of haze pollution transmission in the urban agglomeration of the Yangtze River Economic Belt, identify potential transmission relationships within the urban agglomeration and provide a reference for municipal governments to control haze pollution.

Environmental science research based on air quality models has confirmed that haze pollution has a high degree of spatial contagion. Notably, air pollution in China is spatial autocorrelation [5]. Fan et al and Qin et al simulated air pollution in the Pearl River Delta region using the CMAQ model [6, 7]. Jiang et al and Wang et al modeled haze pollution in the Jing-Jin-Ji area with the GRAPES-CUACE model [3, 8]. Based on the theory of environmental economics, some researchers employed the Extreme Value Boundary Analysis (EBA) model to analyze the spatial correlation and influencing factors of haze pollution in China by combining six socio-economic factors: economy, energy, industry, transportation, population and policy [9]. Zhang used Moran’s I index and LISA scatter plot to explore the spatial correlation of haze pollution [10]. Some scholars have employed quantitative and differential analysis methods to study the spatiotemporal distribution of haze pollution and its relationship with meteorological and climatic factors [11]. Some experts also used the model based on the super relaxation degree (SBM) to estimate the green economic efficiency (GEE) of provinces and cities in Changhe from 2005 to 2018 [12]. Qiang et al analyzed the spatial relationship between urban shrinkage and air pollution in China using the spatial Durbin model (SDM) [13]. They found a negative correlation between urban shrinkage and the increase in air pollution. Lee and Lee analyzed the correlation between air pollution and socioeconomic space in the Seoul Urban Area [14]. Raza et al discussed various issues related to haze in Pakistan, including causes, detection methods, hazardous effects and preventive measures based on ground information [15]. Zhang et al have also investigated air pollution asymmetry from the perspective of spatiotemporal evolution characteristics, and examined the formation, action and impact mechanisms of air pollution asymmetry [16]. Additionally, numerous studies utilize air quality models to investigate regional air pollution transport [17–19].

Nowadays, domestic and foreign scholars mainly focus on urban and regional scale research [20] to explore spatial distribution [21], transmission mechanisms [22] and influencing factors of haze pollution [23]. Moreover, the relationship between air pollution and physical health [24–26]. The existing literature mostly expounds on the transmission characteristics of haze pollution from the overall single dimension. But, this cannot explain the complex superposition attribute in the process of haze pollution transmission. Therefore, analyzing the global conduction characteristics is difficult according to the local conduction relationship. The second is the limitations of research methods. Most scholars use traditional spatial econometric methods to analyze the spillover effect of haze pollution, resulting in research results that can only test the spatial correlation of "adjacent" or "similar" geographical locations.

Therefore, it is important to analyze the relationship between haze transmission between cities from a holistic perspective. This study innovatively utilizes the powerful statistical capabilities of econometrics and social network analysis [27] to empirically investigate the spatial spillover of haze pollution in the Yangtze River Economic Belt urban agglomeration. By doing so, we uncover the characteristics of haze pollution spillover, which serves as empirical evidence to inform haze control strategies in the region. Specifically, this study selects 328 urban meteorological data collection points in the Yangtze River Economic Belt as research samples, takes the city as the main analysis unit, and uses the VAR Granger causality test to study the causal relationship of haze pollution transmission between urban agglomerations after ensuring data stability. To fully understand the spatial correlation influence of haze pollution in urban agglomerations, this study uses the social network analysis method to construct the spatial correlation network of haze pollution in the Yangtze River Economic Belt and investigates its structural characteristics. In addition, this study used the Quadratic assignment procedure (QAP) analysis method to further explore the spatial correlation network of air pollution control efficiency and its influencing factors. According to the characteristics of the cities, the block modeling method divides 36 cities into four types: the net beneficiary type with the highest ability to withstand haze pollution, the net overflow type with the most significant spillover effects, the bilateral overflow type with robust acceptance and spillover capabilities and the broker type acting as an intermediary in haze pollution dynamics. This study uses a network perspective to study the spatial spread of haze pollution, breaking through geographical limitations and providing a new perspective and method for the research of haze pollution. It can better analyze the propagation effect of haze pollution. It is instrumental in providing a more comprehensive understanding of the spatial spillover relationships associated with haze pollution.

## 2. Research technique

### 2.1. Data declaration

**Fig 1:**
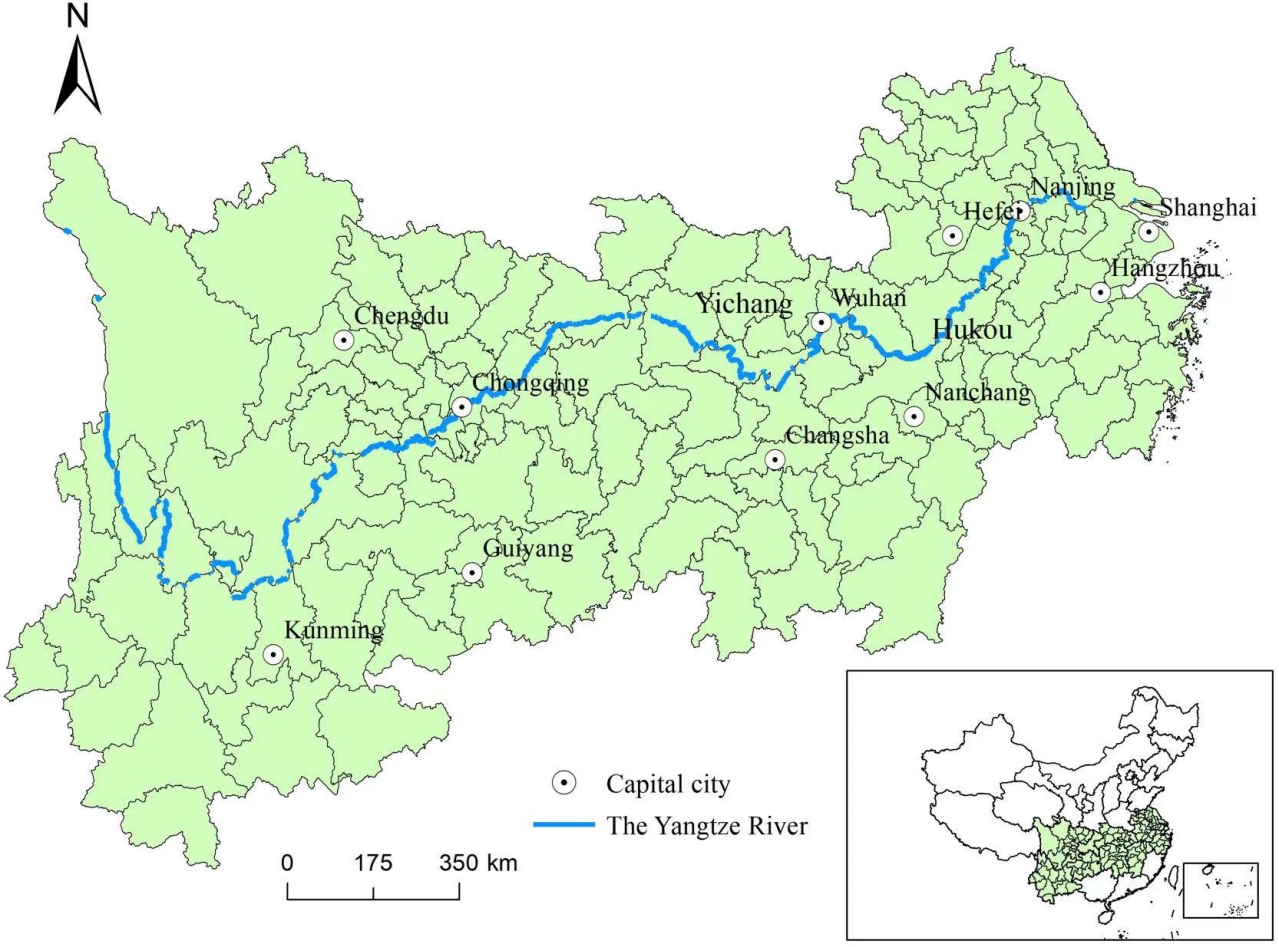
The geographical location of the Yangtze River Economic Belt in, China.

This work selects 36 cities including Shanghai, Chongqing, Nanjing, Wuhan, Suzhou, Hangzhou, Nanchang, Chengdu, Wuxi, Ningbo, Changsha, Hefei, Nantong, Yangzhou, Changzhou, Zhenjiang, Zhoushan, Wuhu, Anqing, Tongling, Chizhou, Jiujiang, Yueyang, Huangshi, Yichang, Luzhou, Yibin, Jingzhou, Panzhihua, Jiaxing, Huanggang, Huzhou, Ma’anshan, Shaoxing, Ezhou and Xianning City as research objects. Among them, are 1 first-level central city, 11 second-level central cities, 15 regional central cities and 9 general cities. Utilize PM_2.5_ monitoring data from January 1, 2021, 00:00 to December 31, 2021, 23:59 in these 36 cities. Summarize the hourly monitoring data and calculate the daily average PM_2.5_ value for each city in 2021 by averaging the values from multiple monitoring stations within the city. The PM_2.5_ data measured at each station in each city and the geographical location of each station are from the Institute of Geographic Sciences and Resources, Chinese Academy of Sciences (http://envi.Ckcest.cn/environment/). The involved provinces and cities of the Yangtze River Economic Belt in this study are shown in Fig 1.

### 2.2. Stability test

For time series, stability requires that the fitting curve obtained by the sample time series can continue along with the existing shape "inertia" or "law" in the future. If the data is unstable, it means that the shape of the sample fitting curve does not have the characteristics of "inertia" or "law" continuation, that is, the curve fitting based on the sample time series to be obtained in the future will be different from the current sample fitting curve.

To analyze the data for each city, we used Eviews software with the Phillips and Perron test (PP test) to verify that the data is a stability time series [28]. In addition, we measured the skewness, kurtosis and standard deviation of the data for each city to give an indirect indication of the stability of the data. Stable data is an essential prerequisite for performing the subsequent Granger causality test. The results are shown in Table 1, which indicate that the data of 36 cities are stable.

**Table 1:** Stability test.

City	Skewness	Kurtosis	S.D.	Mean	PP test
Shanghai	1.076502	1.02275	16.25665	27.5752	-10.563
Chongqing	1.334883	1.1815	21.09582	34.16719	-4.487
Nanjing	1.173489	1.39623	16.7756	30.12556	-8.042
Wuhan	1.559128	3.120119	23.79898	37.0255	-6.558
Suzhou	1.324706	1.869777	17.27911	29.0326	-10.112
Hangzhou	1.1942	2.085336	14.84876	28.41268	-9.03
Nanchang	1.329926	1.640941	19.31384	30.03318	-5.845
Chengdu	1.447304	2.130153	26.85265	39.43362	-6.175
Wuxi	1.10741	1.150122	16.14448	29.55261	-10.481
Ningbo	1.025288	0.666586	12.08955	21.3199	-7.757
Changsha	1.950327	4.556561	30.55841	42.5307	-6.989
Hefei	1.662596	4.277848	20.04706	32.8302	-7.938
Nantong	0.977194	0.375088	18.19815	30.55293	-10.828
Yangzhou	1.348447	2.009093	20.37796	34.03526	-9.386
Changzhou	1.18119	1.560611	20.7932	36.80357	-9.476
Zhenjiang	1.18827	1.447165	21.55862	37.46327	-9.427
Zhoushan	1.278251	1.714922	8.857586	15.16299	-9.805
Wuhu	1.712409	3.856695	20.76293	34.1966	-6.729
Anqing	1.825641	4.728201	21.66318	33.07989	-6.895
Tongling	1.475353	2.857743	18.72082	34.51065	-6.54454
Chizhou	1.885622	4.703543	20.14347	31.86226	-7.3287
Jiujiang	1.483639	2.642048	18.72469	31.37554	-6.87412
Yueyang	1.763412	3.905635	20.16354	35.51928	-6.68374
Huangshi	1.464513	2.931781	19.30623	33.19711	-6.65292
Yichang	1.79138	4.100083	28.95719	38.99697	-5.96381
Luzhou	1.527871	2.102531	27.35555	40.19091	-5.30719
Yibin	1.428262	2.129712	30.36458	43.74968	-6.66353
Jingzhou	1.562805	2.772302	22.49311	35.2966	-6.26729
Panzhihua	1.76014	4.834098	16.01744	30.96419	-9.18466
Jiaxing	1.44063	2.788952	15.48766	26.89417	-10.4956
Huanggang	1.431636	2.747644	18.207	31.71258	-6.54879
Huzhou	1.134027	1.699882	13.03838	24.85813	-9.84416
Maanshan	1.423256	2.402426	19.2861	34.41488	-7.61496
Shaoxing	1.065877	1.179861	14.02984	27.65614	-9.81662
Ezhou	1.640947	3.424344	20.85105	36.84757	-6.8156
Xianning	2.087155	6.056608	19.26336	28.07346	-6.28224

### 2.3. Granger causality

The Granger causality test is a statistical method to identify the causal relationship between two variables. In the research on urban haze pollution, we can use the test to analyze the causal relationship between the degree of urban haze pollution and spatial correlation factors [29]. If the current haze pollution in city *y* can be explained by the past haze pollution in city *x*, it means that the haze pollution in city *x* causes the haze pollution in city *y*.

If the prediction of variable *Y* under the condition of including the past information of variables *X* and *Y* is better than the prediction of *Y* by the past information of Y alone. That is, the variable *X* helps to explain the future variation of the variable *Y*, and the variable *X* is considered to be the Granger cause of the variable *Y*. In more general terms, it means that if the goal is to predict a change in *Y*, plus the prediction of *X* is better than the prediction of only *Y*, then it is said that there is Granger causality between *X* and *Y*.

### 2.4. Social network analysis

Granger causality tests are mainly used to analyze the relational structure of social networks and their properties. The significance of social network analysis lies in its ability to conduct precise quantitative analysis of diverse relationships, providing quantitative tools for constructing intermediate-level theories and testing empirical propositions, thereby bridging the gap between macro and micro levels. The social network analysis used in this study is to illustrate the spatial linkages between cities using network node diagrams with cities as nodes and Granger causality as edges. The study utilizes Ucinet software to analyze the overall structural properties of the network and network clustering.

In social networks, the overall network density is often used to measure the closeness of connections between nodes. The higher the network density, the more closely connected the nodes are. *D* is the network density, *M* is the number of nodes, *N* is the number of actual relationships, and the following formula calculates the network density.


$$D=\frac{N}{M(M-1)}$$ (1)

Network correlation is the degree to which the nodes in a network graph are in contact with each other, the more pathways there are connecting two points, the higher the relevance of the points. Similarly, the more pathways there are between any two points, the higher the cohesion of the whole network. Where *C* is the network correlation, *V* is the number of unreachable pairs of points in the network, and *M* is the number of nodes, the network correlation is calculated as follows.


$$C=1-\frac{V}{\frac{M(M-1)}{2}}$$ (2)

Network efficiency is the degree to which the graph has redundant lines in a directed network graph with a certain number of components. The more paths between two points, the higher the correlation of the points. If there are more paths between any two points, the cohesion of the whole network is greater. *E* is the network efficiency, *R* is the number of redundant lines, and max(*R*) is the maximum number of redundant lines possible. The formula for calculating the network efficiency is shown below.


$$E=1-\frac{R}{\max\left(R\right)}$$ (3)

Network grade is an indicator that reflects the dominant position of a member in the network. *G* is the network grade, *s* is the number of symmetrically reachable points within the network, and max (*S*) is the largest logarithm of symmetrically reachable points within the network.


$$G=1-\frac{s}{\max\left(S\right)}$$ (4)

#### 2.4.1. Network centrality

Network centrality is an index that reflects the position and role of a city in urban agglomeration. Freeman believes that centrality can be expressed by degree centrality, betweenness centrality and closeness centrality [30]. Degree centrality indicates how many points are directly connected to each other. It is divided into in-degree and out-degree in the network, where in-degree is the number of connections between other points and the point, and out-degree is the number of connections between the point and other points. Simply put, the more social connections a point has, the more important that point is. *DC* denotes degree centrality; *n* denotes the number of nodes; *N*_*degree*_ denotes the degree of the node.


$$DC=\frac{N_{degree}}{n-1}$$ (5)

Betweenness centrality is the ability of a node to control the relationship between other pairs of nodes. That is the ability of the point to lie on the shortest path to other points. The higher the intermediate centrality, the more the node is at the center of the network. Let *b*_*jk*_(*i*) be the ability of city *i* to control the relationship between city *j* and *k*, *N* is the number of cities and *BC*_*i*_ represents betweenness centrality, which is calculated as follows.


$$BC_{i}=\sum_{j}^{N}\sum_{k}^{N}b_{jk}(i),(j\neq k\neq i,j<k)$$ (6)

Closeness centrality measures the average length of the shortest path from every node to every other node. In other words, the closer a node is to other nodes, the more central it is. In general, the closeness centrality of a point that needs to be connected by as many points as possible is relatively high. The inverse of the average distance is the closeness centrality. Let *d*_*ij*_ be the shortest distance between city *i* and city *j*, *d*_*i*_ represents the average distance from city *i* to other cities, and *CC*_*i*_ is the closeness centrality. The formula is as follows.


$$\begin{array}{l} d_{i}=\frac{1}{n-1}\sum_{j\neq i}d_{ij}\\ CC_{i}=\frac{1}{d_{i}}=\frac{n-1}{\sum_{j\neq i}d_{ij}} \end{array}$$ (7)

#### 2.4.2. Block modeling analysis

Block modeling is the main method for spatial clustering in social network analysis [31, 32]. The analysis employs the CONCOR algorithm within Ucinet. The process involves both CONCOR classification and the definition of class features. Initially, the city significance correlation spatial spillover relationship table derived from the Granger test analysis is utilized as input. Ucinet’s CONCOR calculation module was configured with a maximum segmentation density of 2 and a convergence criterion of 0.2 [33, 34]. After the computation, 36 cities in the Yangtze River Economic Belt were divided into four types, completing the CONCOR classification. Subsequently, based on the relationships within and between classes as well as the proportions of expected and actual sizes, the four types were designated as net beneficiary type, net overflow type, bilateral overflow type and broker type. It is worth noting that some scholars refer to the four types here as plates or blocks. For the convenience of understanding and reading, we refer to them collectively as types in this article. Following this, the identified types were analyzed. Refer to Table 2 for classification.

**Table 2:** Block modeling classification table.

The proportion of relationships within a location	The proportion of relations received by the location	
	≈0	>0
≥(*g*_*k*_-1)/(*g*-1)	Bilateral overflow type	Net beneficiary type
<(*g*_*k*_-1)/(*g*-1)	Net overflow type	Broker type

#### 2.4.3. QAP regression analysis

In social network analysis, one approach is to study the relationships between relationships. In plain terms, this means the study of correlations and regressions between two square matrices. This approach is called the Quadratic Assignment Procedure. Quadratic assignment procedure (QAP). It compares the similarity of the grid values of two square matrices. The correlation coefficient between the two matrices is given, and the nonparametric test of the coefficient is carried out, which is based on the permutation of the matrix data. Unlike other standard statistical procedures, the values of the matrix are not independent of each other. Therefore, many standard statistical procedures cannot be used to estimate its parameters and statistical tests, otherwise, the wrong standard deviation will be calculated. QAP can measure the relationship between many different values and PM_2.5_ related to this study, which allows us to find many factors that cause haze pollution. The calculation formula is as follows. In the formula, *u* is the matrix of influencing factors. *A* is the spatial correlation matrix of haze pollution in the urban agglomeration of the Yangtze River Economic Belt.


f(A)=f(u) (8)

We use Ucinet software to carry out QAP regression analysis, and the data on urban collaborative development capacity is from November 25, 2021, Xinmin Evening News, that is Yangtze River Economic Belt City Collaborative Development Capacity Index (2021) provided by the organizer. The urbanization rate of each city in the economic belt is derived from the statistical yearbooks of each city.

## 3. Experimental results and analysis

### 3.1 Granger causality test and model establishment

To perform the Granger causality test, first, we need to build a vector autoregressive model (VAR):

$$x_{t}=\alpha_{1}+\sum_{i=1}^{n}\beta_{i}x_{t-i}+\sum_{j=1}^{n}\gamma_{j}y_{t-j}+\mu_{1,t}$$ (9)

$$y_{t}=\alpha_{2}+\sum_{i=1}^{m}\delta_{i}y_{t-i}+\sum_{j=1}^{m}\theta_{j}x_{t-j}+\mu_{2,t}$$ (10)

*m* and *n* are the optimal lag orders determined based on the combined results of the five tests, *LR*, *FPE*, *AIC*, *SC* and *HQ*. The Granger causality test delineates the rejection domain at the 5% significance level. *μ*_1,*t*_ and *μ*_2,*t*_ are random disturbances (or white noise). After estimating this model, test the null hypothesis "*H*_0_: *γ*_1_ *= γ*_2_… *= γ*_*n*_ *=* 0", that is, whether variable *y* helps explain future changes in variable *x*. If *H*_0_ is rejected, *y* is said to be a Granger of *x*. Swapping the positions of *y* and *x* in the regression model can test whether *x* is a Granger of *y* and the causal. The relationship is shown in Table 3. To state the Granger causality of the test, we binarize it. The results are presented in Table 4.

**Table 3:** Granger causality judgment.

Null hypothesis	Result	Null hypothesis	Result	Relationship
*γ*_1_ *= γ*_2_*… = γ*_*n*_ *=* 0	Rejected	*θ*_*1*_ *= θ*_*2*_ *= … = θ*_*n*_ *=* 0	Accepted	*X←Y*
*γ*_1_ *= γ*_2_*… = γ*_*n*_ *=* 0	Accepted	*θ*_*1*_ *= θ*_*2*_ *= … = θ*_*n*_ *=* 0	Rejected	*X→Y*
*γ*_1_ *= γ*_2_*… = γ*_*n*_ *=* 0	Rejected	*θ*_*1*_ *= θ*_*2*_ *= … = θ*_*n*_ *=* 0	Rejected	*X↔Y*
*γ*_1_ *= γ*_2_*… = γ*_*n*_ *=* 0	Accepted	*θ*_*1*_ *= θ*_*2*_ *= … = θ*_*n*_ *=* 0	Accepted	*No*

**Table 4:** Binary matrix of granger causality test.

City	Shanghai	Chongqing	Nanjing	Wuhan	Suzhou	Hangzhou	Nanchang	Chengdu	WuXi	Ningbo	Changsha	Hefei	Nantong	Yangzhou	Changzhou	Zhenjiang	Zhoushan	Wuhu	Anqing	Tongling	Chizhou	Jiujiang	Yueyang	Huangshi	Yichang	Luzhou	Yibin	Jingzhou	PanZhihua	Jiaxing	Huangang	Huzhou	MaAnshan	Shaoxing	Ezhou	Xianning
Shanghai		1	1	1	1	1	1	1	0	0	1	1	0	1	1	1	1	1	1	1	1	1	1	1	1	1	1	1	0	1	1	1	1	1	1	1
Chongqing	1		1	1	1	1	1	1	1	1	1	1	1	1	1	1	1	1	1	1	1	1	1	1	1	1	1	1	1	1	1	1	1	1	1	1
Nanjing	1	0		1	0	1	1	1	0	0	1	1	0	0	0	0	1	0	1	1	1	1	1	1	1	0	1	1	0	1	1	0	1	1	1	1
Wuhan	1	1	1		1	1	1	1	1	1	1	1	1	1	1	1	1	1	1	1	1	1	1	1	1	1	1	1	1	1	1	1	1	1	1	1
Suzhou	1	1	1	1		1	1	1	1	0	1	1	1	1	0	0	0	1	1	1	1	1	1	1	1	0	1	1	1	1	1	1	1	1	1	1
Hangzhou	1	1	1	1	0		1	1	1	0	1	1	0	1	0	0	1	0	1	1	1	1	1	1	1	0	0	1	1	1	1	0	1	0	1	1
Nanchang	1	1	1	1	1	1		1	1	1	1	1	1	1	1	1	1	1	1	1	1	1	1	1	1	1	1	1	0	1	1	1	1	1	1	1
Chengdu	1	1	1	1	1	1	1		1	1	1	1	1	1	1	1	1	1	1	1	1	1	1	1	1	1	1	1	1	1	1	1	1	1	1	1
Wuxi	1	1	1	1	1	1	1	1		0	1	1	1	1	0	0	0	1	1	1	1	1	1	1	1	0	1	1	0	1	1	1	1	1	1	1
Ningbo	1	1	1	1	1	1	1	1	1		1	1	1	1	1	1	0	1	1	1	1	1	1	1	1	1	1	1	0	0	1	0	1	1	1	1
Changsha	1	1	1	0	1	1	1	0	1	1		1	0	1	1	1	1	0	1	1	1	1	1	0	0	1	1	1	0	1	1	1	1	1	0	1
Hefei	1	1	0	1	0	0	1	1	0	0	1		0	0	0	0	0	0	1	1	1	1	1	1	1	1	1	1	0	1	1	0	0	1	1	1
Nantong	1	1	1	1	1	1	1	1	1	1	1	1		1	1	1	1	1	1	1	1	1	1	1	1	1	1	1	0	1	1	1	1	1	1	1
Yangzhou	1	1	1	1	1	1	1	1	1	1	1	1	1		1	1	1	1	1	1	1	1	1	1	1	1	1	1	0	1	1	1	1	1	1	1
Changzhou	1	1	1	1	1	1	1	1	1	1	1	1	0	0		0	1	1	1	1	1	1	1	1	1	1	1	1	0	1	1	1	1	1	1	1
Zhenjiang	1	1	1	1	1	1	1	1	1	0	1	1	0	0	0		1	1	1	1	1	1	1	1	1	1	1	1	0	1	1	0	1	1	1	1
Zhoushan	1	0	1	1	0	1	1	1	0	1	1	1	1	0	0	0		1	1	1	1	1	1	1	1	1	1	1	0	1	1	1	1	1	1	1
Wuhu	1	1	1	1	1	1	1	1	1	0	1	1	0	1	0	0	1		1	1	1	1	1	1	1	1	1	1	0	1	1	1	1	1	1	1
Anqing	1	1	0	1	0	0	1	1	0	0	1	1	1	1	0	1	1	0		0	1	1	1	1	1	0	1	1	1	1	1	1	1	1	1	1
Tongling	1	0	0	1	0	0	1	1	0	0	1	1	0	0	0	0	0	0	1		1	1	1	1	1	0	1	1	0	1	1	0	0	1	1	1
Chizhou	1	0	1	1	0	1	1	1	0	0	1	0	1	0	1	0	1	0	1	0		1	1	1	1	0	1	1	1	1	1	1	0	1	1	1
Jiujiang	1	1	1	0	0	0	1	1	1	1	1	1	1	1	1	1	1	0	0	0	0		1	1	1	1	1	1	1	1	1	1	0	1	1	1
Yueyang	1	1	1	1	0	0	1	1	0	0	1	1	1	0	0	0	1	1	1	1	0	1		1	0	1	1	1	0	1	1	1	1	1	1	1
Huangshi	1	1	1	0	1	1	1	1	1	1	1	1	1	1	1	1	1	1	1	0	1	1	1		1	1	1	1	1	1	1	1	1	1	1	1
Yichang	1	1	1	1	1	1	1	1	1	1	1	1	1	1	1	1	1	1	1	1	1	1	1	1		1	1	1	1	1	1	1	1	1	0	1
Luzhou	1	0	1	1	1	1	1	1	1	1	1	1	1	1	1	1	1	1	1	1	1	1	1	1	1		1	1	0	1	1	1	1	1	1	1
Yibin	1	0	0	1	1	1	0	1	1	1	1	1	1	0	0	0	1	1	1	1	1	1	1	1	1	0		0	0	1	1	1	0	1	1	1
Jingzhou	1	1	1	0	1	1	1	1	1	1	1	1	1	1	1	1	1	1	1	1	1	1	1	1	1	1	1		1	1	1	1	1	1	1	1
Panzhihua	1	1	1	0	1	1	1	1	1	1	1	1	1	1	1	1	1	1	1	1	1	0	1	1	1	1	1	1		1	1	1	0	1	0	1
Jiaxing	0	1	1	1	1	1	1	1	1	0	1	1	1	1	1	1	0	1	1	1	1	1	1	1	1	1	1	1	1		1	1	1	1	1	1
Huanggang	1	1	1	0	1	1	1	1	1	1	1	1	1	1	1	1	1	1	1	1	1	1	1	1	1	1	1	1	0	1		1	1	1	1	1
Huzhou	0	1	1	1	0	1	1	1	0	1	1	1	0	0	0	0	0	1	1	1	1	1	1	1	1	1	1	1	0	0	1		1	1	1	1
Maanshan	1	0	1	1	1	1	1	1	1	0	1	1	1	1	0	0	0	1	1	1	1	1	1	1	1	1	1	1	0	1	1	0		1	1	1
Shaoxing	1	1	1	1	0	0	1	1	1	0	1	1	1	1	0	0	0	0	1	1	1	1	1	1	1	1	0	1	1	1	1	0	0		1	1
Ezhou	1	1	1	0	1	1	1	1	1	1	1	1	1	1	1	1	1	1	1	1	1	1	1	1	1	1	1	1	1	1	1	1	1	1		1
Xianning	1	1	1	1	1	0	1	1	1	0	1	1	1	1	0	1	1	0	0	0	0	1	1	0	1	1	1	1	0	1	1	1	1	1	1	

**Fig 2:**
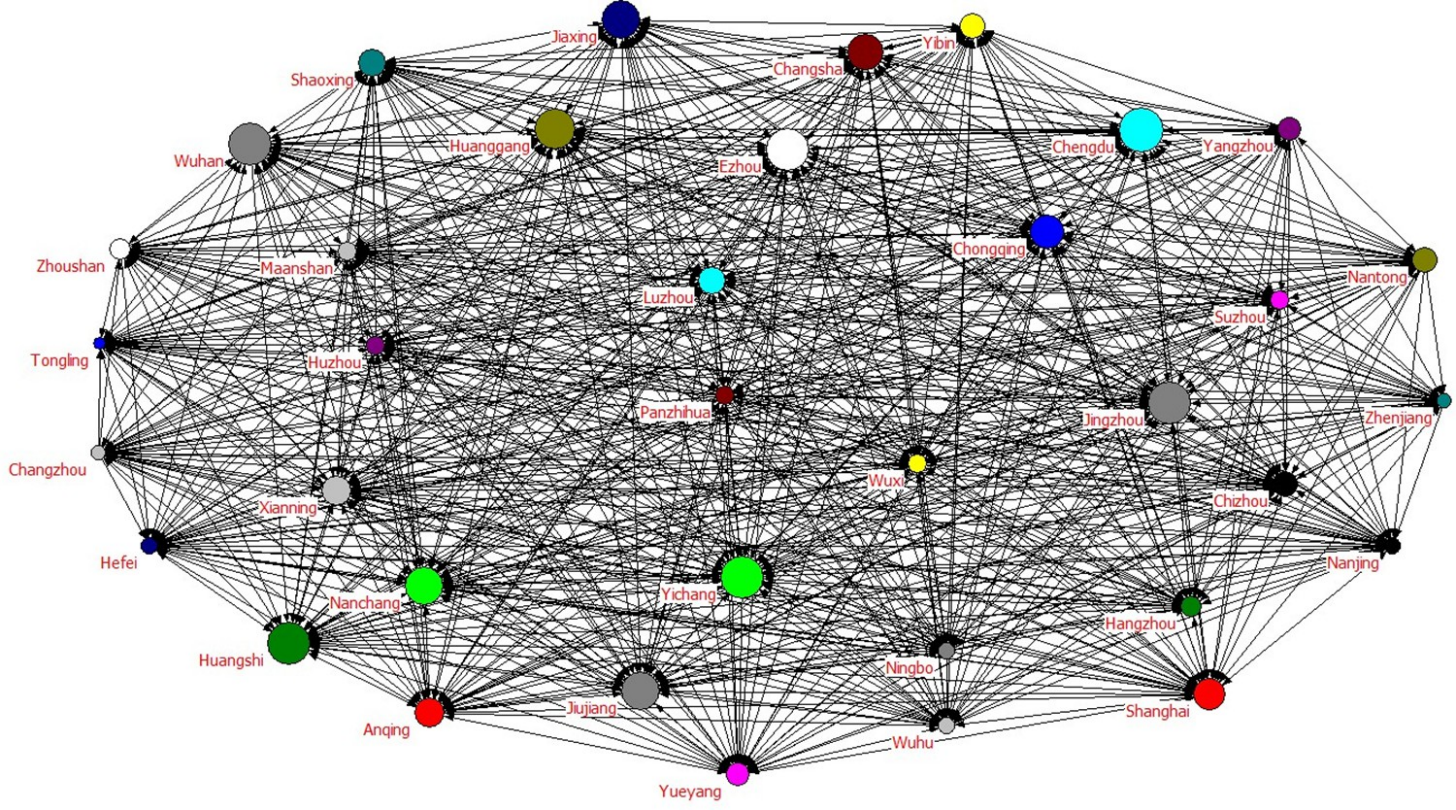
Analysis of the social network.

In Fig 2, the stronger the centrality, the larger the graph representing the city. The arrows in Fig 2 indicate the Granger causality between the two cities, which is obtained according to the Granger causality test above. This graph can show the relationship between each city from the overall view and show the position of a city in the urban agglomeration in the form of images.

Next, continue with the network structure test. Using Eqs (1–4), the network density, correlation, efficiency, and grade of the urban agglomeration network density are 0.8381, 1, 0.0118, and 0 respectively. This suggests that there are no isolated cities in the cluster. This further shows that the relationship between urban agglomerations in the Yangtze River Economic Belt is not a simple spillover or receiving relationship, but a complex spillover relationship. City A can cause pollution to City C through City B, and each city could directly or indirectly affect other cities.

### 3.2 Analysis of network structure and characteristics

Table 5 shows the network centrality of the spatial correlation network of haze pollution in the urban agglomeration of the Yangtze River Economic Belt. Among them, Chongqing, Wuhan and Chengdu City have an out-degree of 35, which is the highest in the Yangtze River Economic Belt urban agglomeration. Furthermore, the results showed that these three cities have the highest impact on haze pollution in other cities. In addition, cities such as Nanchang, Yangzhou, Nantong, Yichang and Ezhou City also have higher out-degree, indicating that haze pollution in these cities has also had a significant impact on haze prevention and control in other cities. The in-degree of Changsha, Yueyang, Huanggang and Xianning City is 35, which is the highest in the Yangtze River Economic Belt urban agglomeration. This proves that these four cities are the cities most affected by haze pollution in the economic belt urban agglomeration. In addition, the in-degree is also relatively high in Nanchang, Chengdu, Hefei, Jiujiang, Jingzhou and Shaoxing City. This also reflects that these cities have a high ability to absorb haze pollution from other cities.

**Table 5:** Centrality analysis.

City	Out-degree	In-degree	Betweenness	In-Closeness	Out-Closeness
Shanghai	31	33	7.249	94.595	89.744
Chongqing	35	28	7.483	83.333	100.000
Nanjing	23	31	1.965	89.744	74.468
Wuhan	35	28	10.347	83.333	100.000
Suzhou	30	24	2.939	76.087	87.500
Hangzhou	25	28	3.491	83.333	77.778
Nanchang	34	34	9.331	97.222	97.222
Chengdu	35	34	11.091	97.222	100.000
Wuxi	29	26	2.620	79.545	85.366
Ningbo	34	19	2.375	68.627	89.744
Changsha	27	35	8.128	100.000	81.395
Hefei	21	34	1.948	97.222	71.429
Nantong	34	25	5.097	77.778	97.222
Yangzhou	34	25	4.414	77.778	97.222
Changzhou	31	19	1.806	68.627	89.744
Zhenjiang	29	20	1.755	70.000	85.366
Zhoushan	28	26	3.393	79.545	83.333
Wuhu	30	25	2.191	77.778	87.500
Anqing	26	33	5.988	94.595	79.545
Tongling	19	30	0.866	87.500	68.627
Chizhou	24	32	5.012	92.105	76.087
Jiujiang	27	34	8.730	97.222	81.395
Yueyang	25	35	4.430	100.000	77.778
Huangshi	33	33	10.208	94.595	94.595
Yichang	34	33	10.420	94.595	97.222
Luzhou	33	27	5.309	81.395	94.595
Yibin	25	33	5.163	94.595	77.778
Jingzhou	34	34	10.675	97.222	97.222
Panzhihua	31	14	2.688	62.500	89.744
Jiaxing	32	33	9.278	94.595	92.105
Huanggang	33	35	9.535	100.000	94.595
Huzhou	25	27	2.817	81.395	77.778
Maanshan	28	28	2.991	83.333	83.333
Shaoxing	25	34	5.365	97.222	77.778
Ezhou	34	32	10.726	92.105	97.222
Xianning	26	35	6.178	100.000	79.515

The city with the highest betweenness is Chengdu, indicating that Chengdu plays the most important role in the haze pollution of the Yangtze River Economic Belt urban agglomeration and is the most important intermediary city in the spatial correlation network. In addition, Wuhan, Huangshi, Yichang, Jingzhou and Ezhou City exhibit high betweenness in urban agglomerations. This indicates that these cities also play a significant mediating role in the urban agglomeration of the Yangtze River Economic Belt. The cities with the highest in-closeness are Changsha, Yueyang, Huanggang and Xianning City, while the cities with the highest out-closeness are Chongqing, Wuhan and Chengdu City, indicating that these cities are not easily controlled and have strong independence. Network centrality testing shows that Chengdu, Chongqing, Wuhan, Nanchang, Yichang, Changsha, Yueyang, Ezhou and Yichang City are located at the center of the spatial correlation network. It is easy to find that most of these are regional or secondary central cities and even primary central cities, which also indicates that haze pollution is directly related to urban development. Therefore, the government departments of cities should develop corresponding management measures. Based on the above analysis, we have concluded that the cities that need to focus on controlling haze pollution are mainly Chengdu, Chongqing and Wuhan City. These three cities have the highest out-degree and Chengdu City has the highest betweenness. Chongqing City has the highest out-closeness. Cities that need to focus on preventing haze pollution are mainly Yueyang, Huanggang, Xianning and Changsha. These four cities have the highest In-degree and In-closeness.

### 3.3 Block modeling results and analysis

To further analyze the role and location of each city in the urban agglomeration of the Yangtze River Economic Belt, we use the CONCOR iterative convergence method in Ucinet software to divide the urban agglomeration into four types. The first is the net beneficiary type. This type of city is characterized by the fact that it receives relationships from both other types and members of its type. But it sends more relationships to others than to others. However, it tends to receive more relationships from other types than it sends. The second type is the net overflow type. As the name suggests, it is akin to the net beneficiary type, but with more relationships sent to other members. The third type is the bilateral overflow type, which both sends relations to other types and receives relations from other types. However, its members have more relationships with each other. The fourth type is the broker type. Its characteristic is that it has established more receiving or spillover relationships with other types of cities.

**Fig 3:**
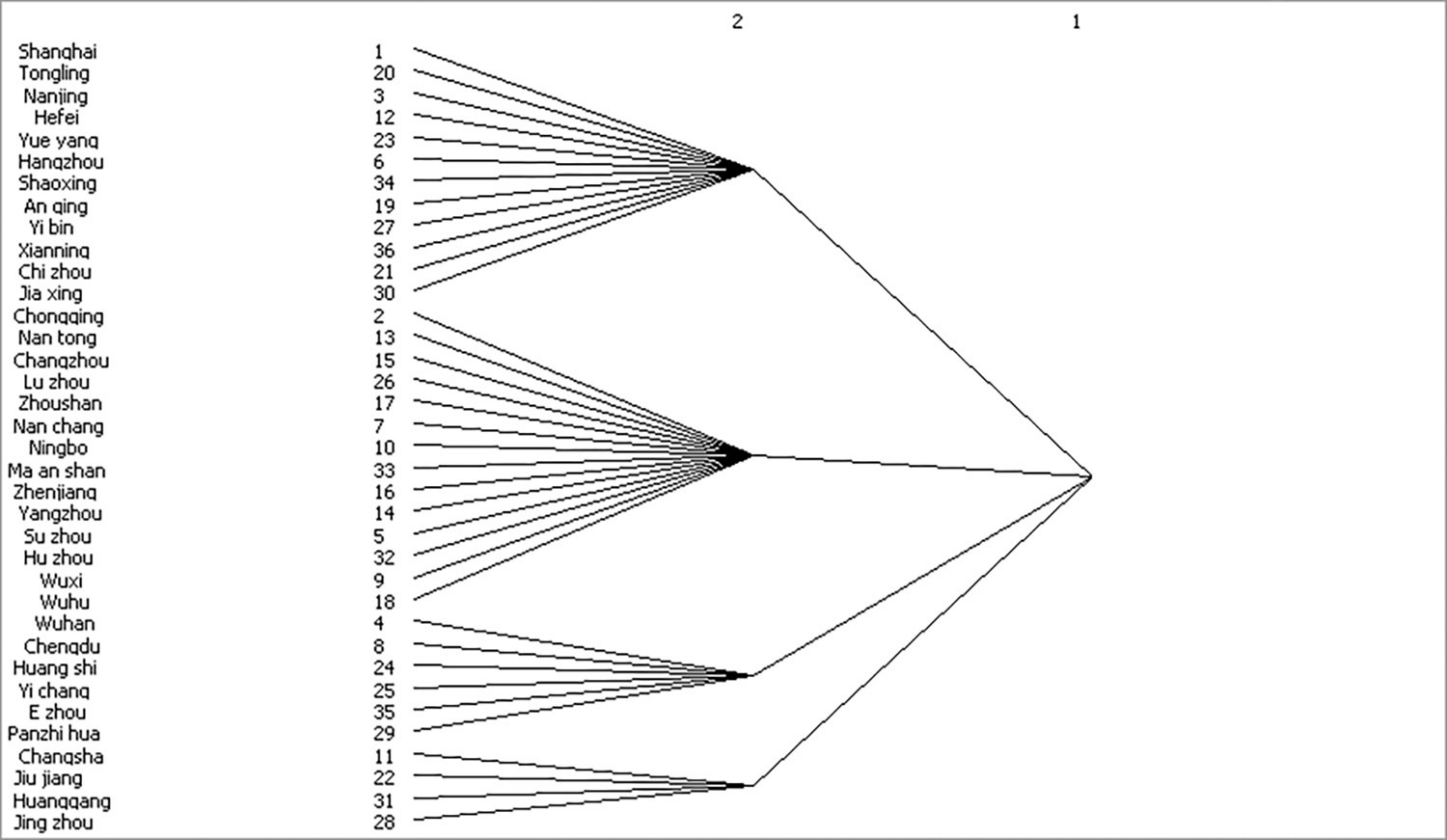
Block modeling dendrogram.

By analyzing Fig 3 and Table 6, we can see clearly that the 36 cities are divided into four groups. The first city group is Shanghai, Tongling, Nanjing, Hefei, Yueyang, Hangzhou, Shaoxing, Anqing, Yibin, Xianning, Chizhou and Jiaxing City. The second group involves Chongqing, Nantong, Changzhou, Luzhou, Zhoushan, Nanchang, Ningbo, Maanshan, Zhenjiang, Yangzhou, Suzhou, Huzhou, Wuxi and Wuhu City. The third contains Wuhan, Chengdu, Huangshi, Yichang, Ezhou and Panzhihua City. The fourth group includes Changsha, Jiujiang, Huanggang and Jingzhou City. To facilitate subsequent type classification, we refer to groups one to four as type one to type four. Upon further investigation, it is found that out of a total of 1,059 relationships, there are 289 relationships between cities within the same type and 770 relationships between different types. This indicates a significant spatial correlation spillover between the types. In the first type, the proportion of actual internal relationships is 36.76%, which exceeds the expected proportion of 31.43%. In the type, the total number of spillovers and benefits are 302 and 191 respectively, and the number of spillovers and benefits are 280 and 191 respectively. The proportion of actual internal relationships in the second type is 32.49% less than the expected proportion of internal relationships is 37.14%. In concrete terms, the total spillover and benefit are 356 and 434, and the benefit and spillover relationship outside the type are 293 and 215 respectively. The actual proportion of internal relations in the third type is 12.38%, which is lower than the expected proportion of internal relations of 14.29%. The total number of spillover and beneficiary relationships within this type is 174 and 202, respectively, and the total number of beneficiary and spillover relationships outside this type is 177 and 149, respectively. The actual internal relationships for type four, at 9.92 percent, were higher than the expected internal relationships of 8.57 percent. And its total overflow and benefit are 138 and 121 respectively, in which 109 relationships benefit from outside the type and 126 relationships spill out of the type. According to the data, compared with types two, three and four, the relationship of type one’s spillovers is far greater than the benefit relationship outside the type, and type one receives the relationship of other modules as well as the relationship of its members. Therefore, we divide type one into the net overflow type. Similarly, compared with types three and four, the number of off-type benefit relations of type two is much larger than the off-type spillover relations, and it receives the relations of other modules as well as its members. So, we divide the type into the net beneficiary type. The remaining bilateral overflow type and broker type are selected in types three and four. Moreover, Table 6 demonstrates that the benefit relationship or spillover relationship outside the type, or the total benefit relationship or total spillover relationship of type three are all greater than that of type four. Even the intra-type relationship of type three is higher than that of four. This shows that type three is a "middleman" position among all types, and the cities in type three are also in the most important position for urban masses to transmit and receive. So, we divide types three and four into broker type and bilateral overflow type.

**Table 6:** Type data obtained from the study as well as the results.

Type	The number of cities	Receive relationship		send relationship		Expected internal relationship ratio	Actual internal relationship ratio	Type categories
		Inside the type	Outside the type	Inside the type	Outside the type			
Ⅰ	12	111	191	111	280	31.43%	36.76%	Net overflow
Ⅱ	14	141	293	141	215	37.14%	32.49%	Net beneficiary
Ⅲ	6	25	177	25	149	14.29%	12.38%	Broker
Ⅳ	4	12	109	12	126	8.57%	9.92%	Bilateral overflow

According to the above analysis, we can obtain the density matrix of each type. We take the data 0.8381 of the network density test in the above network structure as the cut-off point between types 0 and 1 in the image matrix. Thus, further analysis of the spatial relationship of the four types of urban agglomeration. If the density of a certain type exceeds 0.8381, it indicates that the city of that type has a concentrated trend, and this value is set to 1; Otherwise, set it to 0.

It can also be seen from Table 7 that types I to Ⅲ are the main receiving types of haze pollution from others. From that, we know that the three types are also the main source cities of pollution in urban agglomerations. Therefore, we can speculate that the haze pollution transmission and reception relationship between these cities in types I to Ⅲ is intricate. These cities both create and spread pollution. It is an important observation object of haze pollution in the urban agglomeration of the Yangtze River Economic Belt.

**Table 7:** Density matrix and image matrix of haze pollution in urban agglomerations.

Type	Density matrix				Image matrix			
	Ⅰ	Ⅱ	Ⅲ	Ⅳ	Ⅰ	Ⅱ	Ⅲ	Ⅳ
Ⅰ	0.841	0.482	0.875	0.979	1	0	1	1
Ⅱ	0.982	0.758	0.857	1	1	0	1	1
Ⅲ	0.986	0.988	0.833	0.958	1	1	0	1
Ⅳ	0.917	0.911	0.583	1	1	1	0	1

### 3.4 The results of QAP analysis

According to the QAP regression analysis structure presented in Table 8, both the unstandardized and standardized regression coefficients for urban collaborative development capacity are 0.000098 and 0.098054, respectively, both of which pass the significance test at the 0.1 level. This finding suggests a close relationship between haze pollution in the urban agglomerations of the Yangtze River Economic Belt and the cities’ coordinated development abilities. The greater the difference in the collaborative development ability between cities, the stronger the spillover ability of haze pollution. This also reflects that in the urban agglomeration of the Yangtze River Economic Belt, cities with a strong ability to assist the development of the Yangtze River Economic Belt have a stronger ability to spill haze pollution. The unstandardized and standardized regression coefficients of the urbanization rate are -0.51409 and -0.140803 respectively, both of which also pass the 0.1 significance level test. It also shows that the greater the urbanization difference between cities, the stronger the spillover ability between cities. It is easy to think of the relationship between urban agglomerations from the table. The higher the level of urban development and the higher the urbanization rate, the stronger the haze pollution capacity of the city. This shows that the city does not have good control of pollution while developing. As a result, haze pollution is positively correlated with urban development.

**Table 8:** QAP regression analysis.

Variables	Un-stdized coefficient	Stdized coefficient	Significance	Proportion As Large	Proportion As Small
Intercept	1.057780	0.000000	0.000000	0.000000	0.000000
Coordinated development	0.000098	0.098054	0.039000	0.039000	0.962000
Urbanization	-0.514090	-0.140803	0.014000	0.986000	0.014000

### 3.5 Chapter analysis

Based on the above research, we can analyze the key cities in the Yangtze River Economic Belt urban agglomeration through network relationships. The research results indicate that there is a close relationship between haze pollution in the urban agglomeration of the economic belt, and haze pollution is closely transmitted between cities. Chongqing, Wuhan and Chengdu are the three cities with the most severe pollution spillover to other cities and are the main culprits of pollution to other cities in the Yangtze River Economic Belt urban agglomeration. Some cities play a crucial role in transmission. Such as Wuhan, Huangshi, Yichang, Jingzhou and Ezhou. At times, the transmission of haze can even have a greater impact than directly causing pollution. So, these cities can all be the key focus of environmental protection in future urban construction. Yueyang, Huanggang, Xianning and Changsha have outstanding haze reception capabilities. Corresponding measures should be taken to prevent haze.

This reminds us that in the process of promoting urban development, the connection between urban development and environmental protection cannot be ignored. Although some small cities may not have a strong ability to generate pollution themselves, the spread of haze between cities is complex and intricate. Small cities may also become centers of pollution transmission, and their role may not be as direct as creating pollution. However, overall, their role in urban agglomerations may not be inferior to directly creating pollution. Therefore, while developing cities, we cannot ignore these potential cities. We need to adhere to the concept of green development and take the path of sustainable development. Balanced growth of cities and environmental protection is attainable only through scientific planning and effective management. Urban development and environmental protection are closely related, and we should always remember the importance of environmental protection while promoting urban development. In addition, we also need to strengthen environmental cooperation and exchange between cities, share experiences and technologies, and jointly promote the development of environmental protection. Only through international cooperation and exchange can we better address environmental issues between cities and promote their sustainable development.

## 4. Conclusion

This study aims to examine the spatial correlation effects of haze pollution in the urban agglomeration of the Yangtze River Economic Belt from a network perspective. By analyzing the spatial correlation network of haze pollution in the economic belt urban agglomeration, we have gained a deeper understanding of the relationships between urban agglomerations and the transmission and reception of haze pollution. Furthermore, analysis of the impact of urban coordinated development capacity and urbanization rate enhances our understanding of the interplay between urban development and haze pollution.

The article first conducted a unit root test to verify the stationarity of the data. After confirming the stability of the data, the Granger causality test was used to analyze the causal relationship between cities, and the intangible haze transmission relationship was visualized through social network analysis. We performed a centrality analysis to explore the network structure of urban agglomerations and their potential haze transmission characteristics. Furthermore, the implementation of block modeling analysis facilitated the classification of the 36 cities into four distinct groups, each assigned unique roles within the network. Finally, the study utilized QAP regression analysis to investigate the influence of collaborative development capacity and urbanization rate on urban pollution levels within the Yangtze River Economic Belt.

The results indicate that: A pronounced spatial correlation exists within the spatial network of PM_2.5_ in the Yangtze River Economic Belt urban cluster. The haze transmission relationships are not singular. Chengdu, Chongqing, Wuhan, Nanchang, Yichang, Changsha and Yueyang City are situated at the core of the spatial network, with more incoming and outgoing connections. 36 cities can be divided into four groups: the first group includes Shanghai, Tongling, Nanjing, Hefei, Yueyang, Hangzhou, Shaoxing, Anqing, Yibin, Xianning, Chizhou and Jiaxing City. They are the Net overflow role. The second group of cities includes Chongqing, Nantong, Changzhou, Luzhou, Zhoushan, Nanchang, Ningbo, Ma’anshan, Zhenjiang, Yangzhou, Suzhou, Huzhou, Wuxi and Wuhu City. They are playing the role of ’net beneficiary’ in the network. The third group of cities includes Wuhan, Chengdu, Huangshi, Yichang, Ezhou and Panzhihua City. They are located on the ’Bilateral overflow’ type. The fourth group of cities includes Changsha, Jiujiang, Huanggang and Jingzhou City. They are the Broker role. Each group has distinct functional characteristics and linkage effects in the network. QAP regression analysis showed that the relationship between haze pollution and urban collaborative development capacity was significant, as well as haze pollution and urbanization rate. The results of this study provide valuable references for urban government departments in formulating effective management measures, assisting them in better controlling the nexus between urban development and pollution.

For the research of this experiment, we propose suggestions for the future development of cities: Mountains of gold and silver are not as valuable as green waters and lush mountains. The Yangtze River Economic Belt is an important region of China and an important ecological barrier. While striving to develop urban construction in the Yangtze River Economic Belt, we must also pay attention to the impact on the environment. Firstly, through the results of this research, we can focus on pollution control in Chongqing, Wuhan and Chengdu, the three cities with the strongest spillover capabilities. Secondly, cities such as Changsha, Yueyang, Huanggang and Xianning City should strengthen the protection of environmental pollution. Chengdu, Wuhan, Huangshi, Yichang, Jingzhou and Ezhou City are the cities that play the greatest mediating role in urban agglomerations. Relevant government departments can take corresponding environmental protection measures to address this loophole. When formulating relevant policies, government departments should also consider the location of cities in the network and their spatial spillover effects. The third is to establish a compensation mechanism between cities in the Yangtze River Economic Belt urban agglomeration. Different cities should bear different responsibilities in the joint governance of haze pollution. These cities with high levels of urbanization should establish stable financial environmental protection expenditures or haze pollution control funds to compensate cities with low levels of urbanization that suffer from haze pollution. Urban governance is a science and first-class cities need first-class governance. The problems in urban governance are complex and diverse, and many affairs require the coordination of relevant departments in urban governance, as well as the effective participation of social forces. This puts higher demands on promoting the scientific development of governance systems and avoiding inefficiencies caused by multiple government departments. The fourth is to take the comprehensive demonstration zone of China’s new urbanization as an opportunity, upgrade the industrial structure, and encourage the transfer of high-polluting secondary industries to high-value-added service industries [34]. In my opinion, the government can carry out work on haze pollution prediction in the future, and haze prevention and control are to prevent before cure. Relevant departments can study from this direction to carry out haze control work. In recent years, China’s urban ecological environment construction has made significant progress, with significant improvements in ecological protection, environmental governance and green space rates. The construction of park systems has also made significant progress. However, the construction of the ecological environment is always on the way. We must not forget our original intention and adhere to sustainable development.

Research Outlook: In the process of block modeling research, we unexpectedly found part of the content: The first group is primarily located in Jiangsu, Anhui, Zhejiang and Jiangxi Provinces, situated on the right side of the economic belt. Similarly, the second group is mainly found in Jiangsu, Zhejiang, Anhui and Jiangxi Province, also on the right side of the economic belt. The third group, situated in Hubei, Sichuan and Hunan Province, occupies the middle and left areas of the economic belt. Lastly, the fourth group is primarily distributed in Hunan, Jiangxi and Hubei Province, located in the middle of the Yangtze River Economic Belt. This intriguing relationship between spatial characteristics, such as haze, and the geographical location within the Yangtze River Economic Belt warrants further investigation as a potential focal point for future studies.
